# Early Upper Limb Function in Infants Under Three Months: Associations with Shoulder Biomechanics and General Movement Patterns

**DOI:** 10.3390/pediatric17060131

**Published:** 2025-12-03

**Authors:** Lucía Fernanda Flores-Santy, Daniela Celi-Lalama, Juan Pablo Hervás-Pérez

**Affiliations:** 1 MOVS Research Group, Pontificia Universidad Católica del Ecuador, Quito 170525, Ecuador; 2 Faculty of Health Sciences—HM Hospitals, University Camilo José Cela, Urb. Villafranca del Castillo, Villanueva de la Cañada, 28692 Madrid, Spain; jphervas@ucjc.edu; 3 School of Physiotherapy, Faculty of Medical, Health and Life Sciences, International University of Ecuador UIDE, Quito 170411, Ecuador; dacelila@uide.edu.ec

**Keywords:** General Movements Assessment, Denver II, fine motor development, shoulder range of motion, Kinovea

## Abstract

Early identification of neurodevelopmental trajectories is essential for timely intervention in infancy. While joint mobility is often seen as an indicator of motor capacity, its link to early functional performance remains unclear. This study examined whether active shoulder range of motion and the quality of spontaneous movement quality relate to early upper limb function in infants under three months. Thirty-two healthy infants participated in a cross-sectional assessment. Video recordings analyzed with the General Movements Assessment classified movements as Fidgety or Writhing. Fine motor performance was evaluated using five items from the Denver II Screening Test. Active shoulder abduction was measured via two-dimensional frontal-plane analysis with Kinovea^®^. Data analysis involved t-tests and Pearson correlations. Results showed that infants with Fidgety movements scored higher on fine motor tests than those with Writhing movements. Shoulder range of motion was slightly higher in infants with Writhing movements, but not significantly. No sex differences were found. Weak, nonsignificant correlations existed between shoulder range of motion and fine motor performance. The findings suggest movement quality, rather than limb mobility, is more connected to early motor function. Combining movement quality assessments with simple tests may improve early detection of subtle neuromotor issues and guide early stimulation strategies.

## 1. Introduction

The initial three months after birth constitute a crucial phase in human neurodevelopment, during which the Central Nervous System (CNS) begins to organize and solidify fundamental sensorimotor patterns that influence subsequent motor capabilities. In neonates and early infants, upper limb movements serve as important indicators of neuromotor maturity, offering insights into the development of the sensorimotor system [1]. Functional actions—such as reaching, bringing the hands to the midline or mouth, and hand opening—are not only developmental milestones but also reflect the maturation of sensorimotor feedback mechanisms, regulation of muscle tone, and joint mobility [2]. These actions depend on adequate biomechanical support from the musculoskeletal system, especially the shoulder joint complex, which is essential for facilitating functional upper limb movements during early stages of development.

Given its proximal position, multidirectional movement capacity, and close integration with trunk and head control, the shoulder joint is vital for early upper limb development [3]. Restrictions in shoulder Range of Motion (ROM) during this crucial developmental period can delay a child’s ability to explore their surroundings, perform midline and hand-to-mouth movements, and acquire essential skills for self-regulation and sensorimotor integration [4,5,6]. While early neurodevelopmental assessments often depend on qualitative observations, the addition of quantitative biomechanical measurements, such as active ROM, can improve functional evaluations by detecting mechanical limitations that may affect motor performance.

Among the established assessment tools for evaluating developmental progress, the Denver Developmental Screening Test II (Denver II) is regarded as a highly valid [7,8] and widely used instrument for identifying potential developmental delays in children from birth to six years old [9,10]. The test assesses four key developmental domains: gross motor, fine motor-adaptive, language, and personal–social skills. Each item is scored based on direct observation of the child’s performance and compared with normative age-based expectations [11]. The tool is commonly used in pediatric clinical settings to identify potential developmental delays and to guide early intervention or follow-up recommendations [12]. Of particular interest is the fine motor-adaptive domain, which is crucial for early upper limb function as it assesses hand coordination and object manipulation, essential skills for environmental interaction and daily tasks [13]. This domain encompasses behaviors such as hand opening, visual tracking, reaching, grasping, and transferring objects between hands, providing important insights into neuromotor and perceptual-motor integration during infancy [14].

Complementary to developmental screening tools such as the Denver II, Prechtl’s General Movements Assessment (GMA) provides a sensitive and non-invasive method to assess spontaneous motor activity in early infancy. General Movements (GMs) are involuntary, whole-body motor patterns that vary in sequence, speed, and amplitude, with their quality indicative of CNS functionality [15,16]. Normal GMs are primarily categorized into two developmental periods: Writhing Movements (WMs), observed from birth to approximately 8–10 weeks post-term, and Fidgety Movements (FMs), which emerge between 9 and 20 weeks post-term [17]. WMs are characterized by smooth, elliptical, and variable in amplitude and speed movements, while FMs involve small-amplitude, moderate-speed movements of the neck, trunk, and limbs [18,19,20]. The detection of abnormal GMs or the absence of FM correlates with an increased risk of cerebral palsy and other neuromotor disorders [21]. GMA is typically performed by analyzing video recordings of infants in a calm, alert state while lying in supine position. Trained evaluators identify movement patterns indicative of normal or abnormal neuromotor development. This method has demonstrated high sensitivity and specificity for predicting cerebral palsy [20].

Recent evidence suggests that during spontaneous movements in early infancy, the increasing correlations of muscular activity, both ipsilateral and contralateral, flexors and extensors, reflect the ongoing development of neuromotor organization and postural control mechanisms that stabilize the head, trunk, and limbs [3]. Consequently, quantifying upper-limb kinematic parameters, such as active ROM during spontaneous activity, may offer clinically relevant insights into neuromotor maturation and the emergence of functional abilities.

Despite the growing interest in early biomarkers for neuromotor development, there is a significant lack of studies that combine biomechanical parameters with standardized behavioral assessments. Most existing research examines either qualitative aspects of spontaneous movement, such as GMA, or quantitative kinematic descriptors independently, without exploring their combined predictive value [22,23,24]. While tools like the Denver II and GMA are well-established for early detection of neurological risks, the role of peripheral shoulder joint mobility in spontaneous and functional motor performance remains underexplored. Specifically, the relationship between quantitative measures of shoulder mobility and the achievement of early upper-limb developmental milestones requires further investigation during this crucial developmental period.

This study investigates the relationship between active shoulder ROM, GMs types, and early upper limb function in infants under three months of age. Specifically, we examine whether increased active shoulder ROM and the presence of normal GMs are associated with improved performance on fine motor-adaptivetasks assessed by the Denver II scale. We hypothesize that infants exhibiting FM will achieve higher scores on the Denver II scale, and that greater active shoulder ROM will be positively correlated with fine motor-adaptiveperformance. This hypothesis integrates neurophysiological and biomechanical perspectives by evaluating whether qualitative movement patterns or joint kinematics better reflect early neuromotor maturation.

## 2. Materials and Methods

### 2.1. Study Design and Ethical Considerations

This study utilized a cross-sectional observational approach to explore the relationships between active shoulder joint kinematics, spontaneous motor patterns, and the development of upper limb function in healthy infants aged from birth to three months of corrected age. Participants were screened to ensure they were at low neurological risk based on clinical assessments and developmental histories. The research adhered to the ethical standards established by the Declaration of Helsinki. Ethical approval was obtained from the Institutional Review Board of the Human Research Ethics Committee at Pontificia Universidad Católica del Ecuador, under protocol number 2019-61-EO. Written informed consent was obtained from the parents or legal guardians of all participants.

### 2.2. Participants

Thirty-two healthy infants (N = 32) were recruited through intentional convenience sampling from pediatric outpatient clinics and early stimulation centers in Quito, Ecuador. Infants were eligible to participate if they were younger than twelve weeks of corrected age at the time of assessment, were born full-term or preterm but presented stable clinical conditions, and had obtained an APGAR score of seven or higher at five minutes. Only infants without known neurological risk factors—such as hypoxic–ischemic encephalopathy, high-grade intraventricular hemorrhage, or significant perinatal complications requiring prolonged intensive care—were included. Additional eligibility considerations required the absence of congenital anomalies, genetic syndromes, neuromuscular or metabolic disorders, as well as orthopedic alterations that could compromise upper-limb mobility. Infants exhibiting acute illness or medical instability during the evaluation were also excluded to ensure the reliability of the assessments.

Although the sampling approach was deliberate, no infants were excluded based on behavioral fluctuations during data collection. Following standardized procedures for both the GMA and the Denver II, evaluations were conducted only when infants were in an alert–calm state. If an infant became irritable, drowsy, or overstimulated, assessments were temporarily paused and resumed once the infant returned to optimal behavioral conditions. This approach ensured the validity and reliability of recordings and prevented post hoc exclusions due to transient state changes. Behavioral regulation affected only the timing of assessments, not the final sample composition.

The final sample included 20 males and 12 females. Corrected ages ranged from 1.0 to 12.5 weeks (mean ± SD: 9.9 ± 3.0 weeks), while postmenstrual ages ranged from 31.6 to 40.3 weeks (mean ± SD: 37.8 ± 2.1 weeks). This interval covers the developmental window spanning the late WM and FM, considered optimal for qualitative GMA. All infants completed the full evaluation protocol without medical contraindications.

This study was designed as an exploratory, cross-sectional investigation to estimate preliminary parameters for future confirmatory research. In line with methodological guidelines for early or feasibility studies, the sample size was chosen pragmatically while ensuring sufficient precision for effect-size estimation [25,26]. For correlation analyses, sensitivity calculations show that with a sample size of 32, α = 0.05, and power (1–β) = 0.80, the smallest detectable correlation is approximately r ≈ 0.48, indicating a moderate effect [27]. Therefore, the current sample provides adequate sensitivity to identify moderate associations and to estimate parameters with acceptable precision in this early, hypothesis-generating phase.

### 2.3. Procedures

All infants underwent a standardized evaluation protocol consisting of two sequential stages, both conducted on the same day in a quiet, controlled clinical environment to ensure consistency and minimize external influences on motor behavior. The entire evaluation was performed by a pediatric healthcare professional with formal certification and clinical experience in both the GMA and the Denver II Developmental Screening Test.

The first stage involved high-resolution video recording for GMA following Prechtl’s protocol [16,20,28]. Infants were placed in a supine position on a padded mat, in a calm and alert state, and were undressed to enable unobstructed observation of spontaneous motor patterns. A frontal view camera was positioned one meter above the infant to ensure optimal visualization of upper limb movements, especially shoulder abduction. Video recordings were carefully monitored to prevent any tactile or auditory stimulation during this period.

Immediately after the GMA recording, and following a brief rest period if required by the infant’s state, the second stage involved administering selected age-appropriate items from the Denver II Screening Test. These items focused on upper limb use and were assessed individually using standardized materials and procedures. Each item was introduced with appropriate verbal cues and visual stimuli according to the Denver II protocol [29]. If a skill was spontaneously observed during the initial interaction, it was scored as achieved without additional prompting. The evaluator strictly followed the scoring and administration criteria outlined in the Denver II Technical Manual to ensure the reliability and clinical relevance of the functional data collected.

#### 2.3.1. GMA

Spontaneous motor activity was recorded for 30 min with the infant in a supine position following the standardized procedure of the Prechtl’s GMA. Recordings were performed in a controlled clinical environment specifically adapted for infant neurodevelopmental evaluations. The room temperature was kept between 24–26 °C, lighting was soft and indirect to avoid glare, and background noise was minimized. No toys, talking, or handling were allowed during the recording period. Each infant was placed in a supine position on a firm and warm surface, fully awake, in a calm behavioral state, and without clothing to allow clear observation of upper limbs movement. A caregiver and one evaluator were present to maintain comfort and reduce environmental distractions.

Videos were recorded with a high-definition digital camera (1080p resolution, 30 fps) positioned approximately one meter above the infant to provide a full-body overhead view with stable framing. This setup aligns with recommended guidelines for GMA video acquisition, ensuring adequate temporal and spatial resolution for qualitative movement analysis.

The video data were independently reviewed by two pediatric health professionals with formal certification and advanced clinical training in the GMA. Both evaluators were blinded to the infants’ clinical histories and developmental test scores. Each video was assessed using Prechtl’s qualitative framework, with movements classified according to the predominant GMs pattern observed. Infants were subsequently categorized as exhibiting either WM or FM based on movement quality. Those displaying abnormal movement patterns—such as poor repertoire, cramped-synchronized, or chaotic movements—were excluded from the study.

Both evaluators were certified in the Prechtl GMA via the General Movement Trust (G. Einspieler and C. Bosanquet training program) and possessed over ten years of clinical experience in neonatal and infant motor assessments. They were part of the study’s core research team. When disagreements arose between the two primary evaluators regarding movement classification, a senior pediatric neurologist with expertise in early neurodevelopmental assessment was consulted to serve as an adjudicator, ensuring consistency and finalizing consensus ratings. This neurologist supervised the classification process without altering or influencing the independent scores assigned by the primary raters. Interrater reliability was calculated using Fleiss’ Kappa coefficient.

#### 2.3.2. Shoulder Biomechanics—Active Abduction Analysis with Kinovea^®^

Active shoulder abduction was analyzed using digital kinematic analysis based on the same video recordings obtained for the GMA. The analysis focused solely on the frontal plane (coronal view), with the infant in a supine position and the camera positioned directly above the body midline to ensure a clear view of bilateral upper limb movements. Video processing was carried out using Kinovea^®^ (version 0.9.5), an open-source, frame-by-frame 2D motion analysis software validated for clinical, and research use in pediatric motor assessment [30,31,32]. The software enables the manual placement of virtual goniometric arms and the automatic tracking of anatomical landmarks across frames.

For each infant, shoulder abduction was measured by examining each frame to identify when the upper limb reached maximum abduction on either side. The following anatomical reference points were manually marked in the video to define the abduction angle for the tracking: the fulcrum at the acromion process of the shoulder, a reference line drawn vertically from the acromion toward the center of the sternum (representing the midline of the thorax), and the moving arm from the acromion to the lateral epicondyle of the humerus (indicating the upper arm position). The angle between the fulcrum and reference line represented the shoulder abduction angle. All angle data were recorded in degrees and treated as continuous variables.

To ensure consistency, all video analyses were performed by the same evaluator trained in digital motion analysis, who was blinded to the infants’ clinical information and GMA/Denver II classifications.

Previous validation studies have demonstrated that Kinovea^®^ offers high reliability and accuracy for two-dimensional motion analysis in comparison to three-dimensional motion-capture systems. Reported intraclass correlation coefficients (ICCs) are generally ≥ 0.90, and angular measurement errors range from approximately ±1° to ±3°, confirming its appropriateness for clinical and pediatric kinematic applications [33,34]. This level of precision supports the reliability of the current shoulder abduction measurements obtained early.

#### 2.3.3. Upper Limb Function—Denver II Developmental Screening Tool

Upper limb function (ULF) was assessed using eight developmental items derived from the fine motor-adaptivedomain of the Denver II Developmental Screening Test [11]. These items were selected for their ability to specifically identify early upper limb behaviors related to hand use, coordination, and visual-manual integration in infants younger than three months. The chosen items included: follow to midline, follow past midline, hands together, grasp rattle, follow 180° and regard raisin.

Each item was assessed individually in a quiet, comfortable clinical setting to enhance infant engagement. The evaluation was performed by a pediatric healthcare professional certified in administering the Denver II, who was also trained to observe subtle motor and visual behaviors in early infancy. The assessment was carried out by a pediatric physiotherapist trained in administering the Denver II Developmental Screening Test, following a structured professional workshop and supervised clinical practice. The evaluator possessed over five years of experience in pediatric neurodevelopment and was a member of the research team. All testing sessions were conducted under the supervision of a pediatrician specialized in early neurodevelopment to ensure standardization and reliable scoring.

Both the GMA and the Denver II developmental screening were conducted under similar behavioral conditions. All assessments were performed while infants were alert and calm, following at least a 30 min feeding period to minimize irritability or drowsiness. This approach ensured consistent activation levels in the infants across both evaluations and reduced the influence of transient behavioral or arousal fluctuations on fine motor-adaptive performance.

Following the Denver II standardized protocol, each item was introduced using age-appropriate materials and verbal or visual stimuli as needed. However, if the behavior corresponding to an item was spontaneously observed during initial interaction or observation, no additional prompts were used, in accordance with test guidelines. Each item was scored dichotomously: 1 for achieved (behavior clearly demonstrated during the session), or 0 for not achieved (behavior not observed despite appropriate opportunity and stimulation).

The scores of the six items were then added together to create a combined score, ranging from 0 to 6, where higher scores indicated more advanced or age-appropriate functional use of the upper limbs. This total score served as the main outcome measure of functional development in the analysis.

### 2.4. Statistical Analysis

All statistical analyses were conducted utilizing IBM SPSS Statistics^®^ version 28.0. The dataset was meticulously reviewed for completeness, with data integrity validated through double-entry verification. Normality of continuous variables was evaluated via the Shapiro–Wilk test, while Levene’s test assessed homogeneity of variance. An alpha level of *p* < 0.05 was uniformly applied to all inferential analyses.

#### 2.4.1. Reliability of Measurement Procedures

To evaluate the reliability of GMA classification, all video recordings were independently reviewed by two certified evaluators. The concordance between evaluators was quantified using Cohen’s kappa coefficient, with values exceeding 0.80 indicating excellent inter-rater reliability [35].

#### 2.4.2. Descriptive Statistics

Descriptive statistics were used to summarize the demographic and clinical characteristics of the infant sample. Categorical variables, such as sex and GMA classification (FM or WM), were reported as frequencies and percentages. Continuous variables, including the total ULF score and shoulder abduction angles for both the right and left arms, were presented as means and standard deviations for normally distributed data. These descriptive analyses provided an overall overview of the sample and helped determine appropriate inferential methods.

#### 2.4.3. Group Comparisons Based on Sex and GMA Classification

To examine potential sex-based disparities in upper limb development and biomechanics, independent-samples t-tests were used to compare the Denver II total scores (ranging from 0 to 6) and mean shoulder abduction angles for both sides of male and female infants. Additionally, a paired-samples t-test was conducted to evaluate intra-individual differences between right and left shoulder abduction angles, regardless of sex.

Infants were categorized into two groups based on their GMA: those exhibiting FM and those with WM, according to Prechtl’s classification for this developmental stage. Independent-samples t-tests were used to compare these two groups on biomechanical parameters—specifically, mean shoulder abduction angles on both sides, as well as on functional outcomes measured by the total ULF Denver II fine motor-adaptive score (range 0–6). Differences were evaluated for both statistical significance and clinical relevance. Since the variables were continuous, effect sizes were calculated using Cohen’s d to measure the magnitude of the differences between groups.

#### 2.4.4. Comparison of Right and Left Shoulder ROM

A within-subject comparison of right and left shoulder abduction was conducted to assess potential asymmetries between the upper limbs. Paired-samples *t*-tests were employed for data meeting normality assumptions, whereas the Wilcoxon signed-rank test was utilized for non-parametric data. These statistical analyses facilitated the investigation of unilateral biomechanical differences during spontaneous activity in healthy infants.

#### 2.4.5. Correlation Analysis Between Biomechanical and Functional Parameters

To examine the relations between shoulder biomechanics and the development of upper limb functionality, bivariate correlation analyses were performed using Pearson’s correlation coefficient. The analysis examined the relationship between right and left shoulder abduction angles and the total ULF scores. Furthermore, asymmetry indices (calculated as the absolute difference between right and left shoulder abduction angles) were correlated with ULF scores to assess the influence of inter-limb variability on functional performance.

#### 2.4.6. Multivariate Analysis: Two-Way ANOVA

A two-way analysis of variance was conducted to examine the main and interaction effects of sex and GMA classification on the ULF score. This statistical model was chosen to determine whether these categorical variables exert independent or synergistic influences on functional upper limb development. Prior to performing the ANOVA, assumptions of normality, homogeneity of variances, and independence of observations were verified to ensure the validity of the analysis.

#### 2.4.7. Predictive Modeling: Multiple Linear Regression

To determine the variables significantly associated with upper limb function, a multiple linear regression model was constructed with the ULF score serving as the dependent variable. The independent variables incorporated into the model included right shoulder abduction ROM, left shoulder abduction ROM, GMA classification, and sex. This analytical approach facilitated the concurrent assessment of biomechanical and neurodevelopmental factors influencing functional motor outcomes

## 3. Results

### 3.1. Interrater Reliability

The Fleiss’ Kappa coefficient indicated a statistically significant level of interrater reliability in the classification of GMs video recordings, with a Kappa value of 0.909 (*p* < 0.001), demonstrating an almost perfect agreement among raters.

Data normality was verified using the Shapiro–Wilk test for each group and shoulder variable. For left shoulder abduction, results were W(23) = 0.949, *p* = 0.285 for the FM movement group and W(9) = 0.928, *p* = 0.462 for the writhing movement group. For right shoulder abduction, values were W(23) = 0.930, *p* = 0.111 and W(9) = 0.948, *p* = 0.672, respectively. All *p*-values exceeded 0.05, confirming the assumption of normal distribution across groups.

Homogeneity of variances was confirmed using Levene’s test, which showed F(1,30) = 2.083, *p* = 0.159 for right shoulder abduction, F(1,30) = 2.296, *p* = 0.140 for left shoulder abduction, and F(1,30) = 0.995, *p* = 0.327 for the Denver II fine motor-adaptive scores. These findings validated the assumptions of normality and homoscedasticity, supporting the use of parametric statistical methods for subsequent analyses.

### 3.2. Descriptive Statistics

A total of 32 infants under three months of age were included in the analysis. Of these, 62.5% were male and 37.5% were female. According to Prechtl’s GMA, 71.9% demonstrated FM, indicative of typical neuromotor development, while 28.1% exhibited WMs, which are associated with atypical developmental trajectories.

As detailed in Table 1, the mean shoulder abduction ROM was 39.27° (SD = 16.03) on the right side and 40.70° (SD = 15.17) on the left side. The mean ULF score, derived from the fine motor-adaptive domain of the Denver II scale, was 4.59 (SD = 1.16), with a range spanning from 3 to 6 points. The average asymmetry in shoulder abduction across subjects was 11.64° (SD = 8.18), presenting considerable individual variability.

### 3.3. Upper Limb Function by General Movement Type

A comprehensive comparison between infants classified with FM and WM revealed robust and clinically relevant differences in early ULF. Infants exhibiting FM attained notably higher scores on the fine motor-adaptive domain of the Denver II assessment (M = 4.96, SD = 1.07) compared to those with WM (M = 3.67, SD = 0.87) with a mean difference of 1.29 points (95% CI: 0.47–2.11) and a large effect size (Cohen’s d = 1.02), highlighting the clinical magnitude of the association. These findings suggest that the presence of FM may reflect a more developed neuromotor organization, facilitating more effective engagement in goal-directed upper limb activities.

In contrast, infants with WM exhibited numerically greater shoulder abduction ranges bilaterally. Specifically, the mean right shoulder abduction was 45.83° (SD = 21.06) in the WM group and 36.71° (SD = 13.28) in the FM group, with a mean difference of −9.13° (95% CI: −21.76° to 3.51°). Similarly, the mean left shoulder abduction was 46.82° (SD = 10.74) in the WM group and 38.31° (SD = 16.15) in the FM group, with a mean difference of −8.51° (95% CI: −20.48° to 3.46°). Although these differences did not reach statistical significance (*p* > 0.05), the moderate effect sizes (Cohen’s d ≈ −0.57) suggest potential biomechanical trends that merit further investigation with larger samples.

### 3.4. Sex Differences

The comparison between sexes utilizing independent samples t-tests indicated no statistically significant differences in shoulder ROM or ULF scores (*p* > 0.05 for all tests). Although females demonstrated marginally higher ULF scores (mean = 5.00) compared to males (mean = 4.35), this difference was not statistically significant (*p* = 0.127). Effect sizes were uniformly small (Cohen’s d < 0.3), implying an absence of clinically meaningful sex-related differences in early upper limb development.

### 3.5. Shoulder Symmetry and Within-Subject Differences

The paired-samples analysis indicated no statistically significant difference between the abduction angles of the right and left shoulders within subjects (t(31) = −0.56, *p* = 0.577). The mean difference observed was −1.43°, slightly favoring the left shoulder. The correlation between the two sides was strong (r = 0.581, *p* < 0.001), and the effect size, as measured by Cohen’s d, was negligible (d = −0.10), thereby supporting the clinical expectation of symmetrical motor development during early infancy.

### 3.6. Correlation Analyses

Pearson correlation analyses revealed no significant relationships between shoulder abduction angles on both sides and the total ULF Denver II score. Specifically, the right shoulder abduction exhibited a weak, negative correlation with Denver II performance (r = −0.032, *p* = 0.861), while the left shoulder abduction showed a weak, positive correlation (r = 0.084, *p* = 0.648). Shoulder asymmetry (Abduction Difference) was also not associated with functional outcomes (r = −0.044, *p* = 0.811). All correlation coefficients are shown in Table 2.

### 3.7. Two-Way ANOVA: Effects of GMA and Sex

**Figure 1:**
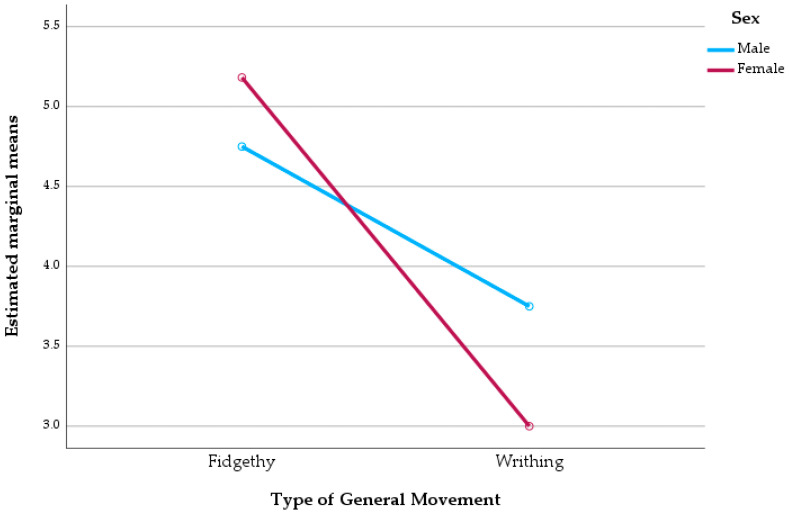
Estimated marginal means of ULF score by type of GM and sex.

A two-way ANOVA was performed to examine the main and interaction effects of sex and GMs type on ULF. The analysis indicated a significant main effect of GM types on ULF Denver II scores (F(1,28) = 7.425, *p* = 0.011), with a partial eta squared (η^2^p) of 0.210 denoting a moderate effect size, suggesting that infants exhibiting FM demonstrate higher levels of functional capacity. Conversely, there was no significant main effect of sex (*p* = 0.787), nor was there a significant interaction between sex and GM type (*p* = 0.320). The model accounted for approximately 22% of the variance in upper limb function, as indicated by an adjusted R^2^ of 0.220 (Figure 1).

### 3.8. Multiple Linear Regression

**Figure 2:**
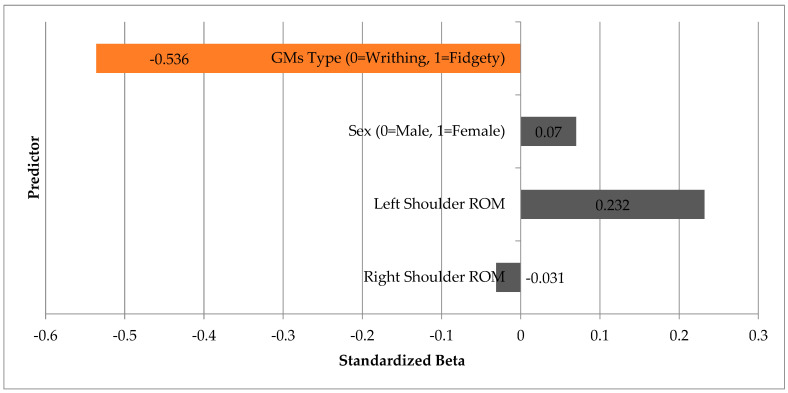
Standardized Coefficients (Beta) from the Multiple Linear Regression Model.

A linear regression analysis was performed to assess the predictive value of shoulder ROM (right and left), sex, and GMA type on ULF scores. The overall model was statistically significant (F(4,27) = 3.056, *p* = 0.034) and explained approximately 21% of the variance in functional scores (adjusted R^2^ = 0.210). The sole significant predictor was GMA type (β = −0.536, *p* = 0.006) (Figure 2). Neither shoulder ROM nor sex appeared as significant contributors to the model. These findings underscore the importance of qualitative assessment of motor patterns over purely biomechanical measurements in infants under three months of age.

## 4. Discussion

This study aimed to examine whether active shoulder ROM and GMs types are associated with early upper limb function in infants under three months. Based on our hypothesis, we expected that infants exhibiting FM would demonstrate higher fine motor-adaptive scores and that greater active shoulder ROM would be positively correlated with functional performance. Our results partially supported this hypothesis: GMA classification emerged as a strong predictor of functional outcomes, while shoulder ROM showed no significant association with early upper limb function.

### 4.1. General Movement Patterns and Early Upper Limb Function

The strong association between GMA classification and Denver II performance reinforces extensive evidence supporting FM as a hallmark of typical neuromotor maturation. Previous studies have demonstrated that FM reflects the increasing integration of cortical–subcortical networks and the refinement of central pattern generators supporting emerging voluntary control [2,36]. Our results align with this literature: infants exhibiting FM showed significantly higher fine motor-adaptive scores, with a large effect size, indicating more efficient early sensorimotor integration.

This finding also supports the clinical relevance of GMA as a sensitive early biomarker of neurological integrity [37,38]. Consistent with the foundational work of Prechtl and Hadders-Algra [39,40], the present study confirms that the quality, variability, and complexity of spontaneous movements provide more accurate indicators of early brain function than isolated biomechanical measures.

### 4.2. Shoulder ROM and Early Motor Function

Active shoulder ROM did not show a significant correlation with fine motor-adaptive performance. Although infants with WM showed slightly higher abduction ROM, this trend was not statistically significant. Importantly, as indicated by developmental motor research, larger joint excursions in early infancy may indicate reduced postural control or physiological hypotonia rather than a functional advantage [41,42]. This perspective may explain our findings: increased ROM in infants with poorer movement quality could result from diminished neuromotor constraints rather than enhanced biomechanical capacity.

This interpretation is consistent with studies demonstrating that early spontaneous movements are meaningful only when integrated with adequate central coordination, rather than when considered as isolated joint actions [43,44,45]. Therefore, central movement organization, rather than the extent of limb excursion, appears to be the primary determinant of early functional development [46,47,48].

### 4.3. Bilateral Symmetry, Interlimb Coordination, and Sex Differences

Shoulder ROM was largely symmetrical between limbs, consistent with developmental patterns driven by subcortical pattern generators that promote bilateral coordination in early infancy [46,47,48]. Interlimb correlations were moderate, supporting the expectation of symmetry during this period.

The influence of sex was examined both independently and in interaction with GM classification. Although females showed marginally higher ULF Denver II scores on average, sex did not significantly affect functional outcomes nor interact with GM patterns. These findings indicate that sex-related differences in early neuromotor function are likely subtle and context-dependent, with central motor patterning playing a more prominent role in early upper limb behavior [49,50,51,52].

### 4.4. Strengths of the Study

A key strength of this study is the integration of two complementary approaches: qualitative neurophysiological assessment using GMA and quantitative kinematic measurement of shoulder ROM, a combination rarely explored in early infancy [3]. Additional strengths encompass rigorous evaluator training, standardized environmental and behavioral conditions during recordings, and a clinically homogeneous cohort of healthy infants, which collectively enhance internal validity.

### 4.5. Clinical and Public Health Perspectives on Early Motor Assessment

From a clinical standpoint, these findings highlight the importance of prioritizing qualitative assessments of spontaneous movement in early infancy. The GMA is a reliable, non-invasive screening tool capable of distinguishing typical from atypical neuromotor trajectories even prior to the emergence of voluntary movements. Clinicians should interpret joint mobility with caution, as increased ROM may indicate neuromotor inefficiency rather than enhanced capacity.

From a public health perspective, incorporating GMA alongside simple functional tools like the Denver II can enhance early detection networks in primary care settings. Integrating structured motor observations into routine well-baby visits may facilitate the early identification of atypical developmental patterns, particularly in low-resource environments.

### 4.6. Future Research Directions

Longitudinal studies are needed to examine whether GM patterns and early ROM profiles predict later motor, cognitive, or functional outcomes. Incorporating three-dimensional motion analysis or inertial sensor technology may provide a more comprehensive understanding of early upper limb biomechanics. Expanding future samples to include infants at neurological risk will enhance the ecological validity and clinical translational potential of this line of research.

### 4.7. Limitations

Despite promising findings, some methodological limitations related to motion analysis should be acknowledged. Specifically, the use of two-dimensional (2D) kinematic analysis, though practical, cost-effective, and suitable for clinical settings, limits assessment to the frontal plane and cannot capture scapulohumeral rhythm, thoracic contributions, or out-of-plane movements. These factors are crucial for understanding the coordinated biomechanics of the shoulder complex and may have resulted in a partial view of true joint excursion. Additionally, subtle scapular rotations or trunk compensations could not be quantified with the current setup. Future research should utilize three-dimensional motion capture or multicamera systems to provide a more comprehensive analysis of early upper-limb kinematics.

Several additional limitations must be considered. Although the sample size was adequate for preliminary analysis, it restricts statistical power and generalizability. The Denver II assessment was limited to five items within the fine motor-adaptivedomain, which may reduce the granularity of functional characterization. Furthermore, although the Denver II demonstrates acceptable reliability in Latin American populations [9,10], its partially observer-dependent scoring may introduce subtle measurement variability despite standardized administration.

Shoulder ROM was assessed during spontaneous movement rather than structured tasks, which may introduce variability due to differences in infant arousal, positioning, or spontaneous motor intent. Behavioral state was standardized by evaluating infants in a calm, alert condition following feeding; however, no formal neonatal behavioral scale (e.g., Brazelton NBAS) was applied. This procedure minimized arousal-related variability while maintaining clinical feasibility.

Finally, the cross-sectional design precludes conclusions about developmental trajectories. Longitudinal studies are needed to determine whether early GMs quality and biomechanical parameters predict later motor and functional outcomes.

## 5. Conclusions

This study demonstrates that GM quality, specifically the presence of typical FM, is a more consistent and reliable indicator of early upper limb function in infants under three months of age than active shoulder ROM. In line with our hypothesis, infants exhibiting FM achieved significantly higher fine motor-adaptive scores on the Denver II, whereas shoulder ROM showed no meaningful association with functional performance. These findings challenge the assumption that greater joint amplitude directly reflects better early motor ability and instead highlight the primacy of central neuromotor organization over peripheral joint excursion during this developmental period.

Our findings highlight that the quality, variability, and coordination of spontaneous motor patterns are more crucial for functional development than overall limb mobility. This emphasizes the clinical importance of combining qualitative assessments, such as the GMA, with specific functional tests to improve early developmental monitoring. Notably, even with a limited set of fine motor-adaptive items, qualitative differences in motor organization showed strong links to functional outcomes, reinforcing early movement quality as a sensitive marker of neurodevelopmental integrity.

Future longitudinal studies are necessary to assess the predictive validity of early GMA and functional performance regarding future developmental trajectories. Such research could elucidate the relationship between early neuromotor patterns and emerging motor skills, thereby enhancing early detection of neuromotor impairments and informing the development of more tailored early intervention and stimulation strategies.

## Figures and Tables

**Table 1:** Descriptive Statistics for biomechanical and functional variables in healthy infants under three months of age *.

	N	Min	Max	Mean	Standard Deviation
Right Shoulder Abduction Range of Motion	32	18.10	83.12	39.2741	16.02961
Left Shoulder Abduction Range of Motion	32	10.00	74.95	40.7007	15.16738
Upper Limb Function Denver II score	32	3	6	4.59	1.160
Abduction Difference	32	0.00	33.81	11.6428	8.17678
N valid	32				

* Values are presented as mean, minimum, maximum, and standard deviation. Abduction Difference represents the absolute difference between right and left shoulder abduction range of motion. Upper Limb Function Denver II score corresponds to the sum of fine motor-adaptive items assessed.

**Table 2:** Pearson correlation matrix between shoulder abduction biomechanics and early fine motor-adaptive performance in infants *.

		Right Shoulder Abduction Range of Motion	Left Shoulder Abduction Range of Motion	Upper Limb Function Denver II Score Fine Motor- Adaptative	Abduction Difference
Right Shoulder Abduction Range of Motion	Correlación de Pearson	1	−0.032	0.025	0.581
	Sig. (bilateral)		0.861	0.893	<0.001
	N	32	32	32	32
Left Shoulder Abduction Range of Motion	Correlación de Pearson	0.581	0.084	−0.082	1
	Sig. (bilateral)	<0.001	0.648	0.655	
	N	32	32	32	32
Upper Limb Function Denver II score Fine Motor-Adaptative	Correlación de Pearson	−0.032	1	−0.044	0.084
	Sig. (bilateral)	0.861		0.811	0.648
	N	32	32	32	32
Abduction Difference	Correlación de Pearson	−0.025	−0.044	1	−0.082
	Sig. (bilateral)	0.893	0.811		0.655
	N	32	32	32	32

* Values represent Pearson correlation coefficients (r) and two-tailed significance levels (*p*) for associations between biomechanical variables (right and left shoulder abduction range of motion and interlimb asymmetry) and functional performance (Denver II fine motor-adaptive score). Significant correlations are indicated at *p* < 0.05. Abduction Difference represents the absolute difference between right and left shoulder abduction angles.

## Data Availability

The dataset generated and analyzed during the current study is publicly available in the Figshare repository at: https://doi.org/10.6084/m9.figshare.29923388.

## Abbreviations

The following abbreviations are used in this manuscript:

GMs	General Movements
FMs	Fidgety Movements
WMs	Writhing Movements
ULF	Upper Limb Function
GMA	General Movements Assessment
ROM	Range of Motion
CNS	Central Nervous System
